# Production of α-Glycerylphosphorylcholine in Fermented Roots, Tubers, and Fruits

**DOI:** 10.3390/foods13193085

**Published:** 2024-09-27

**Authors:** Timothy J. Tse, Farley Chicilo, Daniel J. Wiens, Jianheng Shen, Javier Anleu Alegria, Young Jun Kim, Ji Youn Hong, Jae Kyeom Kim, Eui-Cheol Shin, Martin J. T. Reaney, Youn Young Shim

**Affiliations:** 1 Department of Food and Bioproduct Sciences, University of Saskatchewan, 51 Campus Drive, Saskatoon, SK S7N 5A8, Canada; farley.chicilo@usask.ca (F.C.); daniel.wiens@usask.ca (D.J.W.); jis956@mail.usask.ca (J.S.); jaa339@mail.usask.ca (J.A.A.); martin.reaney@usask.ca (M.J.T.R.); 2 Department of Food and Biotechnology, Korea University, 2511 Sejong-ro, Sejong 30019, Republic of Korea; yk46@korea.ac.kr (Y.J.K.); nutrigenomics@korea.ac.kr (J.K.K.); 3 Department of Food Regulatory Science, Korea University, 2511 Sejong-ro, Sejong 30019, Republic of Korea; khjy1025@korea.ac.kr; 4 Department of Food Science, Gyeongsang National University, 501 Jinju-daero, Jinju 52828, Republic of Korea; eshin@gnu.ac.kr; 5 Prairie Tide Diversified Inc., 102 Melville St., Saskatoon, SK S7J 0R1, Canada

**Keywords:** α-glycerylphosphorylcholine, fermentation, root, tuber, fruit, ethanol, value-added products

## Abstract

Vegetables and fruits, high in starch and sugars, are promising substrates for bioethanol production, but can also yield valuable nootropic compounds, such as α-glycerylphosphorylcholine (α-GPC). This compound is a known cognitive enhancer that works by increasing the release of acetylcholine, a neurotransmitter essential for learning and memory. In this study, select root and tuber crops, as well as fruits, were subjected to *Saccharomyces cerevisiae* fermentation to observe the co-production of ethanol and α-GPC. The ethanol yields from these substrates were comparable to those from wheat (var. AC Andrew), ranging from 30.44 g/L (beet) to 70.04 g/L (lotus root). Aside from ethanol, α-GPC was also produced, with purple top turnip yielding 0.91 g/L, the second highest concentration after wheat (used as a reference), which produced 1.25 g/L. Although α-GPC yields in the tested substrates were lower than those from cereal grains (e.g., wheat and barley), a noteworthy observation was the production of methanol in many of these substrates. Methanol was detected in all feedstocks except wheat, with concentrations ranging from 0.10 g/L (cassava) to 1.69 g/L (purple top turnip). A linear regression analysis revealed a strong correlation between methanol and α-GPC content (R^2^ = 0.876; slope = 0.52), suggesting a potential link in their biosynthetic pathways. These feedstocks not only proved effective as substrates for bioethanol production, but also showed potential for generating value-added compounds such as α-GPC. This dual-purpose potential presents new market opportunities for producers by leveraging both biofuel and nootropic compound production. Furthermore, the observed relationship between methanol and α-GPC production warrants further investigation to elucidate the metabolic pathways involved.

## 1. Introduction

The global demand for energy continues to increase due to economic expansion and population growth [1]. Due to environmental concerns, there has been a shift towards renewable energy sources (e.g., bioethanol). Bioethanol is commonly produced using starch- and/or sugar-rich [2] substrates that are often used for food production (e.g., wheat, sugarcane, etc.) [3]. These feedstocks are often chosen due to their carbohydrate content, as well as their availability in their respective geographical regions.

Similarly, roots and tuber crops are not only used for food [4] but have also been cultivated for various industrial applications. Many of these root and tuber crops, such as potatoes, are an important commodity in developed and developing countries, as they are not typically a globally traded commodity, and they produce large quantities of dietary energy and stable yields under growth and soil conditions in which other crops might fail [5]. Potatoes have higher carbohydrate yields compared to cereals, with ~85% of the biomass of a potato plant being edible, compared to 50% in cereals [5]. In Europe, potato yields are also the second highest per unit area of production (22,840 kg ha^−1^) among the top five crops (i.e., wheat, sugar beet, maize, potatoes, and barley) [6]. In addition, the starch content for many roots and tubers (16–24%) makes these crops attractive for bioethanol production [7]; however, these crops are not typically used as fermentation substrates that afford valuable products (e.g., α-glycerylphosphorylcholine) [8]. 

Meanwhile, fruits, such as bananas and plantains, are underutilized as a potential growth medium for yeast, despite its high carbohydrate and essential nutrient content [9]. However, the fermentation of agri-waste products (e.g., banana peels), including spoiled products, has been investigated as a feedstock source for biofuel production. These fruits are readily available in tropical and subtropical regions and are relatively inexpensive and accessible for their use in fermentation. They also posses high carbohydrate contents ranging between 22.8 and 31.9 g (per 100 g) [10]. 

Several of the substrates described in this study could be utilized in biofuel production, and offer alternative feedstock solutions based on their high starch yield and sustainability to grow in marginal lands (e.g., poor soils or low rainfall climates). Typical biofuel feedstocks in developed countries include wheat, barley, sugar beet, and corn. From these feedstocks, ethanol yields have ranged between 59.6 g/L and 72.1 g/L in wheat [11], 68.0 and 78.5 g/L in barley [12], and 50.6 and 72.0 g/L in oats [12], depending on the cultivar. Similarly, sugar beet has observed ethanol yields of 52.3 g/L, using immobilized yeast [13], and corn reported ethanol yields of 74.6 g/L [14]. Nonetheless, the potential to produce value-added compounds during bioethanol fermentation served as the motivation for this study, which focused on examining various co-products produced from carbohydrate-rich roots, tubers, and selected fruits.

*Saccharomyces cerevisiae* is commonly used for commercial bioethanol fermentation. During the fermentation process, a variety of value-added co-products can be produced alongside ethanol, including organic acids, glycerol, and α-glycerylphosphorylcholine (α-GPC) [11,12,15,16,17]. α-GPC is of commercial interest due to its cognitive and health applications [18] and is also a dietary source of choline. This compound is a precursor for the neurotransmitter acetylcholine and serves as a cognitive enhancer in the treatment of neurological diseases (e.g., Alzheimer’s) [18], and has also been used to enhance brain function and muscle strength [19]. This compound can be produced chemically [20] or enzymatically [21]; however, chemically derived α-GPC cannot be used as a food ingredient due to the presence of toxic substrates and catalysts [22]. In addition, enzymatic processes, using phospholipases, are expensive and can be challenging due to their long reaction times [23,24,25]. Currently, global market projections for nootropic supplements are predicted to surpass USD 10 billion by 2025 [26]. Therefore, the production of this valuable compound using rapid and inexpensive fermentation methods can potentially provide producers with additional sustainable revenue streams, while also addressing commercial market demands. Interestingly, the investigation of α-GPC production via fermentation has been relatively limited compared to other compounds, highlighting the need for further research in this area. The production of this nootropic compound through industrial fermentation processes (e.g., biofuels) presents a potential opportunity for producers to enhance revenue by isolating and purifying these compounds for new market entries. For example, previous studies utilizing common bioethanol feedstock, specifically wheat [11], barley [12], and oats [12], have reported α-GPC yields ranging from 1.24 g/L to 1.68 g/L for wheat [11], 0.84 g/L to 1.81 g/L for barley [12], and 0.62 g/L to 0.88 g/L for oats [12], with concentrations varying depending on the cultivar. 

Although some of the feedstocks investigated in this study are used in bioethanol production outside of North America, the concentration of α-GPC and other organic solutes have not been extensively studied. Many roots, tubers, and fruits contain elevated amounts of carbohydrates, making them potential candidates for *Saccharomyces cerevisiae* fermentation. The successful fermentation of these substrates could open up their use for new applications, including biofuels and nootropic value-added components. Therefore, the primary purpose of this study was to survey commonly available vegetables and fruits as potential substrates for α-GPC production, via *Saccharomyces cerevisiae* fermentation. 

## 2. Materials and Methods

### 2.1. Materials

Ten vegetables and two fruits (Table 1) were purchased from the Save-On-Foods supermarket (Saskatoon, SK, Canada). In addition, AC Andrew, a commonly grown wheat cultivar, was also fermented as a reference substrate. The skin and seeds were removed prior to the gelatinization, saccharification, and fermentation of the substrates. Commercial enzymes (α-amylase, glucanase, xylanase, and glucoamylase) and urea were acquired from Terra Grain Fuels, Belle Plaine, SK, Canada. Meanwhile, commercial yeast (Bio-Ferm^®^ XP; Lallemand Biofuels & Distilled Spirits, Milwaukee, WI, USA) was acquired from PoundMaker Agventures (Lanigan, SK, Canada). These reagents were used in previously published studies [11,12], and the volumes were kept identical to ensure reproducibility and to compare fermentation results with these previous studies. 

### 2.2. Gelatinization

The vegetables and fruits were roasted at 180 °C for 2.5 h to gelatinize the substrates by degrading the cell wall, making the starch and fibres more accessible for saccharification. After roasting, the substrates were cooled to room temperature and minced using a Black & Decker food processor (Model FP3300) (Middleton, WI, USA) equipped with the grater blade attachment. Approximately 260 g (wet weight) of each substrate was mixed with 300 mL of boiled water (100 °C) and incubated at 130 °C for 1 h using a VWR Constant Temperature Oven (Model 1350GM; Mississauga, ON, Canada), with stirring every 15 min. Meanwhile, wheat grain (var. AC Andrew) was milled to a coarse flour using a Glen Mills Type C/11/1 tabletop grinder/disc mill (Model 1350GM; Mississauga, ON, Canada), and was similarly gelatinized with boiled water (36% *w*/*v*) and incubated under the same conditions as the other substrates. 

### 2.3. Saccharification and Fermentation

The saccharification of all substrates was accomplished via enzymatic hydrolysis. Initially, ⍺-amylase (0.2%, *v*/*v*) was added to the mash, followed by incubation at 80 °C for 60 min. A 1:3 mixture of glucanase:xylanase (*v*/*v*) was then added to the mash (0.04%, *v*/*v*) and further incubated at 55 °C for 30 min, with stirring every 15 min. Samples were then cooled to 37 °C, followed by the addition of glucoamylase (0.04%, *v*/*v*), yeast (0.1%, *w*/*v*; *Saccharomyces cerevisiae*), and liquid urea (0.04%, *v*/*v*). A gas trap was then fitted onto each fermenter. The final liquified fermentation volume was 250 mL for each sample. Each fermentation broth was incubated at 37 °C until completion after 72 h. An aliquot of 2 mL fermentation broth was collected for ^1^H-NMR spectroscopy analysis at 0, 24, 48, and 72 h to monitor fermentation progression. All fermentations were conducted in duplicate. 

### 2.4. Moisture Content Determination

The moisture content for each substrate was determined to normalize the results of each substrate to a standard 36% (*w*/*v*). Briefly, 5 g of unprocessed substrate (wet weight) was retained to determine moisture content via drying in a VWR Constant Temperature Oven (Model 1350GM; Mississauga, ON, Canada) at 105 °C for 72 h, until the dry weight remained consistent (e.g., within 5% difference). These analyses were conducted in triplicate.

### 2.5. Organic Solutes Determination via ^1^H-NMR Spectroscopy

Samples for ^1^H-NMR analyses were prepared in a similar manner as described by Tse et al. [17] (Table 2). Spectroscopy data collection (Table 2) and analyses were conducted using the TopSpin™ 3.8 software (Bruker BioSpin GmbH, Billerica, MA, USA). Unfortunately, the glucose content of the fermentation mash was not reported, as the use of ^1^H-NMR spectroscopy can have limitations in accurately quantifying the concentration of sugars, due to the “accidental overlap” of the C-H units in the carbohydrate backbone [27]. In addition, glucose measurements in complex solutions (e.g., pH changes) can be difficult [28], as these molecules can undergo mutarotation [29], further inhibiting accurate measurements for glucose, using this method.

### 2.6. Statistical Analysis

Statistical analyses of organic solute content (ethanol, methanol, and α-GPC) were performed using R (version 4.4.0), using the ggplot [30] and dply [31] packages. A Kruskal–Wallis rank sum test followed by Dunn’s post hoc test with Benjamani–Hochberg correction for multiple comparisons was used to identify significant differences in ethanol, methanol, and α-GPC concentrations; wheat (var. AC Andrew) was used as the reference substrate. Meanwhile, linear regression analysis was applied using Microsoft Excel (version 16.84) to identify variables that could affect α-GPC yields. Significant differences were reported at the 95% confidence interval (*p*-value < 0.05).

## 3. Results and Discussion

To compare the vegetable and fruit substrates with previous studies [17,18] and wheat (AC Andrew), the results were normalized to a standard 36% (*w*/*v*) equivalent using each substrate’s moisture content (Table 1 and Equations (1) and (2)). Although the production of other organic acids and solutes (i.e., acetic acid, lactic acid, succinic acid, and glycerol) were also observed (Figures S1–S4), they will not be expanded on, as the primary purpose of this short communication was to investigate α-GPC yields in these feedstocks.

(1) $$Feedstock\;Percent\;\left(\frac{w}{v}\right)=\frac{W\times \left(1-\left(\frac{M}{100}\right)\right)}{V}$$

where *W* = wet weight of feedstock, *M* = moisture content, and *V* = total volume

(2) $$Normalized\;Correction\;Factor=\left(\frac{36\%}{FP\;\%}\right)$$

where *FP* is the feedstock percent (*w*/*v*). The normalized correction factor was then multiplied to each organic solute to estimate its concentration, assuming a 36% (*w*/*v*) feedstock solution.

**Figure 1:**
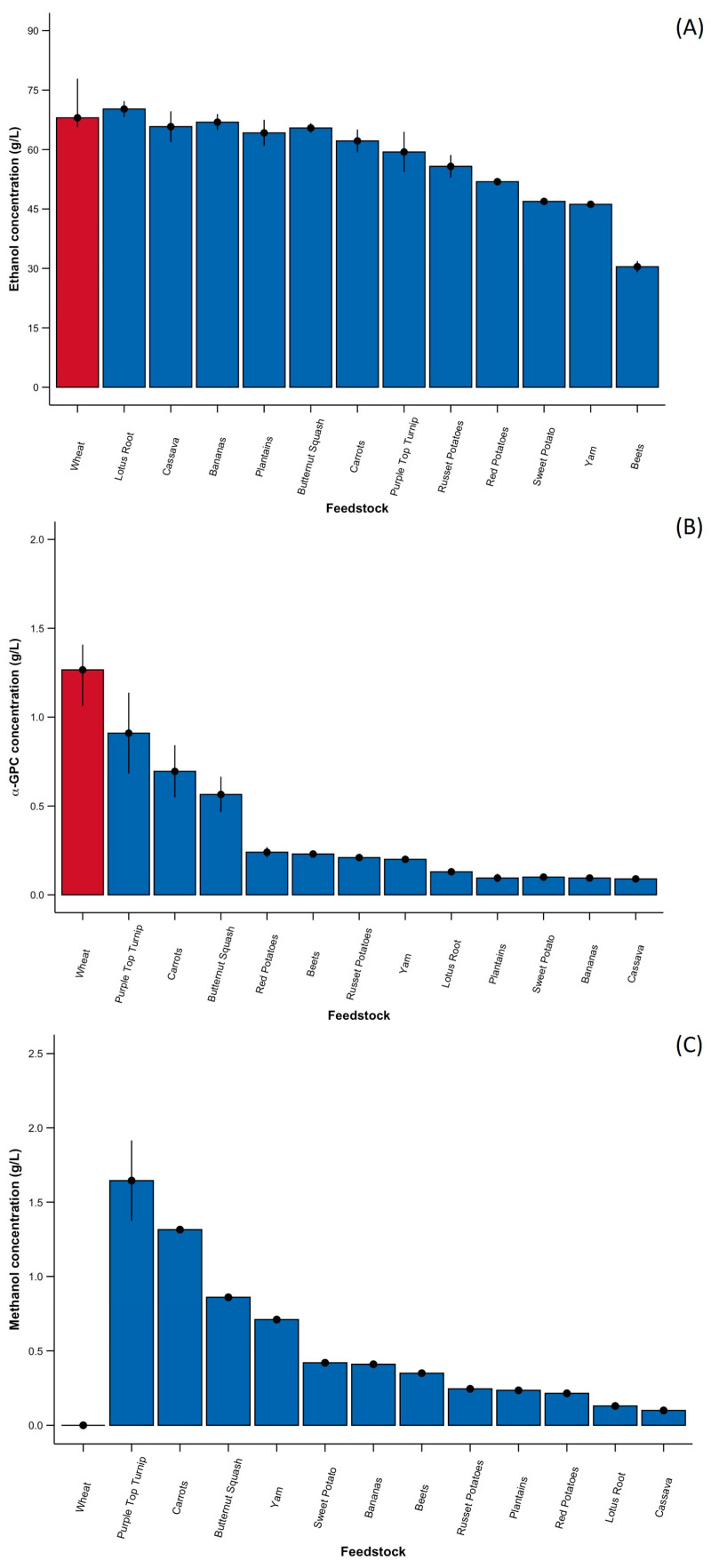
Median ethanol (**A**), α-GPC (**B**), and methanol (**C**) concentrations after 72 h fermentation. Wheat is highlighted in red (on the left-most side) and other substrates are labelled in blue for comparative purposes. Error bars represent the 2.5th and 97.5th percentile for each sample.

The ethanol content (Figure 1A) varied amongst the different substrates, with beet accumulating the lowest amounts (30.44 g/L ± 2.11 g/L) and lotus root resulting in the highest (70.04 g/L ± 5.88 g/L). However, there were no statistical differences in ethanol content among all the substrates (*p*-value > 0.05). The ethanol content was also comparable to typical feedstocks used in biofuel production, such as wheat, barley, and oat [12], suggesting that these substrates could be utilized for bioethanol production. This is likely attributed to the high starch content and accessible sugars of some of these substrates. For example, sweet potato and cassava possess high starch contents, with reported values of 65.8% and 78.0%, respectively, making them viable substrates for bioethanol production [32]. Comparatively, in previous studies, ethanol yields in wheat ranged between 59.6 g/L and 72.1 g/L [11], 68.0 and 78.5 g/L in barley [12], and 50.6 and 72.0 g/L in oats [12], although concentrations were affected based on the cultivar.

Similarly, the accumulation of α-GPC (Figure 1B) varied amongst the substrates, with cassava yielding the least (0.09 g/L) and purple top turnip accumulating the most (1.25 ± 0.35 g/L), aside from wheat, which was used as the reference. The top three substrates that yielded a significantly higher α-GPC content, aside from wheat, were purple top turnip (*p*-value = 0.04), carrots (*p*-value = 0.03), and butternut squash (*p*-value = 0.03). Meanwhile, all other substrates exhibited no statistical differences among each other (*p*-value > 0.05). Regardless, these substrates produced significantly less α-GPC compared to wheat (between 1.24 g/L and 1.68 g/L) [11], barley (between 0.84 g/L and 1.81 g/L) [12], and oats (0.62 g/L and 0.88 g/L) [12]. Although many of these crops have been considered for bioethanol production due to their high yields, there have been comparatively less demand for these substrates for use in food and fodder applications [8]. The lower α-GPC content in roots and tubers could be associated with their decreased phospholipid contents compared to seeds [33]. Cereal grains have relatively high amounts of lysolecithin and lysophosphatidylethanolamine, some of which are present in starch grains. Together, these two lipid classes account for over 90% of wheat phospholipids [33]. The choline content in the root/tuber substrates has also been reported to be lower compared to wheat and other cereal grains [34].

In addition, substrates containing elevated amounts of precursor phosphatidylethanolamine or phosphatidylcholine could potentially result in greater concentrations of α-GPC [35]; however, the α-GPC content appears to be unaffected by its phospholipid content [11]. In addition, the use of phospholipase enzymes could enhance α-GPC production, but was not incorporated into this study. Several of the substrates have been previously investigated for their application in bioethanol production due to their high starch contents [35], including cassava [36], sweet potato [37], potato [38], sugar beet [39], and yam [38]. Pre-treatment methods such as milling and acid/alkaline hydrolysis could enhance fermentation outcomes [40]; however, this was not investigated in this study. Likewise, developing a method to extract α-GPC from the fermentation broth could be challenging due to its water solubility, and should be considered once an ideal feedstock and optimized fermentation conditions have resulted in profitable yields.

Finally, methanol content (Figure 1C) was minimal in all fermentations, with concentrations ranging from 0.10 ± 0.01 (cassava) to 1.69 ± 0.42 g/L (purple top turnip). Methanol was not produced in the wheat fermentation. Similarly to the α-GPC results, only purple top turnip (*p-*value = 0.02), carrots (*p*-value = 0.02), and butternut squash (*p*-value = 0.04) exhibited statistical differences in methanol content. All other substrates did not exhibit any significant differences among each other (p-values > 0.05). The production of methanol in *Saccharomyces cerevisiae* fermentation can occur in a variety of pathways, including (1) the hydrolysis of pectin during fermentation [9]; (2) via the hydrolysis of cellulose, followed by the conversion of polyols into methanol [41]; or (3) amino acid metabolism such as the conversion of methionine to *S*-Adenosyl-L-methionine (SAM), which can then be demethylated, resulting in the release of methanol [42]. Nonetheless, the increased presence of methanol observed in fermentations of purple top turnip and carrot might arise from pectin, a component of the cell walls of plants, composed of acidic sugar-containing backbones with neutral sugar-containing side chains [43]. Pectin can be distributed in the skin of some vegetables [44], with carrot and potato having observed high amounts of pectin compared to other vegetables [45]. Decreasing pectinase activity [46] or the extraction of the pectin [47] from these substrates prior to fermentation might reduce methanol accumulation. The extracted pectin can also then be used as a commercial emulsifier in food production [47].

**Figure 2:**
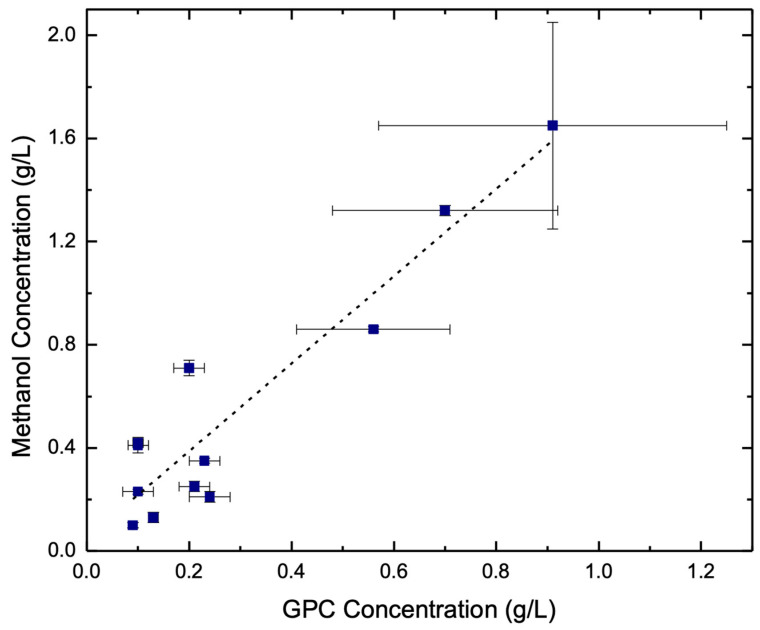
Linear regression analysis suggesting a potential relationship between the presence of methanol and the production of α-GPC in the feedstocks investigated. The blue dots represent observed data points during the experiment with standard deviation for α-GPC (horizontal) and methanol (vertical). Meanwhile, the dashed line represents the regression line, demonstrating a positive linear relationship between these two analytes.

To examine the relationship between α-GPC and other co-produced organic solutes, a linear regression model was applied (Table 3). This model can help reveal relationships between different compounds, thereby providing insight into potential metabolic pathways on how these compounds may interact or influence each other. This analysis revealed a strong relationship between α-GPC and methanol within the fermented substrates (Figure 2; R^2^ = 0.876). Meanwhile, the slope values exhibited in Table 3, suggest the potential rate of change between measured organic solutes, with a negative slope indicating an inverse relationship. For example, a slope of 0.52 between methanol and α-GPC indicates that for every 1 g/L increase in methanol, the α-GPC concentration increased by 0.52 g/L. Altogether, this suggests that these two variables are strongly related (R^2^ = 0.876), but small changes in methanol may not result in large changes in α-GPC (slope = 0.52). The positive correlation between methanol and α-GPC was further visualized as a plot (Figure 2), and suggests that methanol levels can potentially be used to predict α-GPC levels during fermentation.

However, this potential relationship between α-GPC and methanol within the fermented substrates could also be attributed to fermentation from bacteria, which could also impact the production of co-produced organic acids (Figures S1–S4) [48], as well as metabolic processes involving SAM [49]. However, the metabolism of methanol can also potentially improve SAM regeneration by using methanol as a methyl group donor [50]. SAM is an important molecule involved in numerous biological and chemical pathways, acting as a methyl donor for numerous methylation reactions [51]. This compound is involved in the synthesis of choline [52] and phosphatidylcholine synthesis [53], which can then be converted to α-GPC [11]. Therefore, the presence of methanol, by virtue of pectin-containing substrates, could potentially contribute to the production of α-GPC, although further investigation would be needed to test this hypothesis. Nonetheless, the production and purification of α-GPC could potentially create supplemental value.

Although root and tuber crops can have a lower yielding content of some fermentation products compared to cereals and grain seeds, their adaptability to different soil types, low input requirements, and resistance to pests and diseases can make them an attractive alternative for biofuel production [54]. Utilizing damaged or diseased harvests of these crops for biofuel production may also provide an alternative use for crops unsuitable for food use. The production and extraction of these value-added components could provide an additional source of revenue for biofuel producers. However, it is also worth noting that ripeness can affect both carbohydrate and sugar content in the substrate [55]. Therefore, future investigations could also focus on fermenting these feedstocks at various cycles of ripeness, as well as under more controlled conditions, to investigate and optimize potential changes to improve α-GPC yields.

## 4. Conclusions

The production of α-GPC via fermentation has not been widely investigated in the literature. Previous studies have demonstrated the production of this valuable nootropic in wheat, barley, and oat; however, other potential substrates had not been investigated. Therefore, the purpose of this short communication was to survey a variety of readily available root, tuber, and fruit substrates as a potential fermentation feedstock for the production of α-GPC. The results indicated that several of these substrates (e.g., lotus root, cassava, banana, plantains, butternut squash, carrots, purple top turnip, russet potato, and red potato) could obtain comparable ethanol yields to wheat, barley, and oats, which are typical feedstocks for bioethanol production. Meanwhile, the production of α-GPC was observed in all substrates, although in lesser yields than reported in cereal grains (e.g., wheat, barley, and oat). Of all the substrates tested, purple top turnip, carrots, and butternut squash produced the highest yields of α-GPC. Interestingly, these three substrates also produced the highest yields of methanol. Linear regression analysis revealed a strong relationship between α-GPC and methanol, suggesting that these two analytes are strongly related. This potential relationship could be attributed to exogenous bacterial fermentation, as well as metabolic processes involving SAM. Nonetheless, the presence of pectin-containing substrates can result in the production of methanol during fermentation, and could also potentially contribute to the production of α-GPC. The further development of purification technologies to isolate α-GPC could then potentially create supplemental value when applied at an industrial setting (e.g., bioethanol production). With a growing concern regarding the use of food-based substrates for biofuel production, future research could explore the use of damaged or diseased crops for both bioethanol and α-GPC production. This approach could also involve improving and optimizing fermentation conditions under more controlled environments, as well as gaining a deeper understanding into the metabolic pathways involved in α-GPC production.

## Figures and Tables

**Table 1:** Moisture and carbohydrate content for different feedstocks.

Sample	Moisture Content (%)	Carbohydrate Content (per 100 g) *
Banana	75.97 ± 0.65	22.8
Beet	83.82 ± 0.23	9.56
Carrot	87.02 ± 0.27	9.08
Cassava	57.76 ± 2.78	38.1
Butternut Squash	90.64 ± 0.40	11.7
Large Sweet Potato	71.10 ± 0.48	20.1
Lotus Root	85.82 ± 0.33	17.2
Plantain	60.44 ± 0.92	31.9
Purple Top Turnip	92.13 ± 0.26	6.43
Red Potato	82.71 ± 2.82	15.9
Russet Potato	78.42 ± 1.50	17.5
Yam	82.05 ± 0.69	27.9

* Carbohydrate values were retrieved from the U.S Department of Agriculture, Agricultural Research Service [10].

**Table 2:** Chemical shift (ppm) for identified organic solutes via ^1^H-NMR.

Organic Solute	Chemical Shift (ppm)
Ethanol	1.07
Lactic Acid	1.25
Acetic Acid	1.95
Succinic Acid	2.50
Methanol	3.16
α-GPC	3.11
Glycerol	3.45
Pyrazine (internal standard)	8.5
Deuterium Oxide (solvent)	4.7

**Table 3:** Linear regression model (coefficient of determination; RSQ) between different organic solutes produced during fermentations of root and tuber feedstocks.

RSQ			
	Ethanol	GPC	Methanol
Ethanol	------	0.011	0.002
GPC	0.011	------	0.876
Methanol	0.002	0.876	------
**Slope**			
Ethanol	------	0.00	0.00
GPC	4.39	------	1.70
Methanol	1.15	0.52	------

## Data Availability

The original contributions presented in the study are included in the article, further inquiries can be directed to the corresponding authors.

## Supplementary Materials

The following supporting information can be downloaded at: https://www.mdpi.com/article/10.3390/foods13193085/s1, Figure S1: Average acetic acid concentrations, for each feedstock, after 72 h fermentation. Wheat is highlighted in red and other substrates as labelled in blue for comparative purposes, Figure S2: Average lactic acid concentrations, for each feedstock, after 72 h fermentation. Wheat is highlighted in red and other substrates as labelled in blue for comparative purposes, Figure S3: Average succinic acid concentrations, for each feedstock, after 72 h fermentation. Wheat is highlighted in red and other substrates as labelled in blue for comparative purposes, Figure S4: Average glycerol concentrations, for each feedstock, after 72 h fermentation. Wheat is highlighted in red and other substrates as labelled in blue for comparative purposes.
