# Supplementary material for: Production of α-Glycerylphosphorylcholine in Fermented Roots, Tubers, and Fruits
Source: Foods. 2024 Sep 27;13(19):3085. doi: 10.3390/foods13193085 (PMC11475080; doi:10.3390/foods13193085)
Supplement: Supplementary file 1 [file foods-13-03085-s001.zip › foods-3194829-supplementary.pdf]

Supplementary Information: Production of a-Glycerylphosphorylcholine in Fermented Root, Tuber, and Fruit

Timothy J. Tse[1,*], Farley Chicilo[1], Daniel J. Wiens[1], Jianheng Shen[1], Javier Anleu Alegria[1], Young Jun Kim[2], Jae Kyeom Kim[2], Eui-Cheol Shin[3], Martin J. T. Reaney[1,2,4], and Youn Young Shim[1,2,4,*]

1 Department of Food and Bioproduct Sciences, University of Saskatchewan, 51 Campus Drive, Saskatoon, SK, S7N 5A8, Canada

2 Department of Food and Biotechnology, Korea University, Sejong 30019, Republic of Korea

3 Department of Food Science, Gyeongsang National University, 501 Jinju-daero, Jinju 52828, Republic of Korea

4 Prairie Tide Diversified Inc., 102 Melville St., Saskatoon, SK, S7J 0R1, Canada

* Correspondence: timothy.tse@usask.ca (T.J.T.), younyoung.shim@usask.ca (Y.Y.S.)

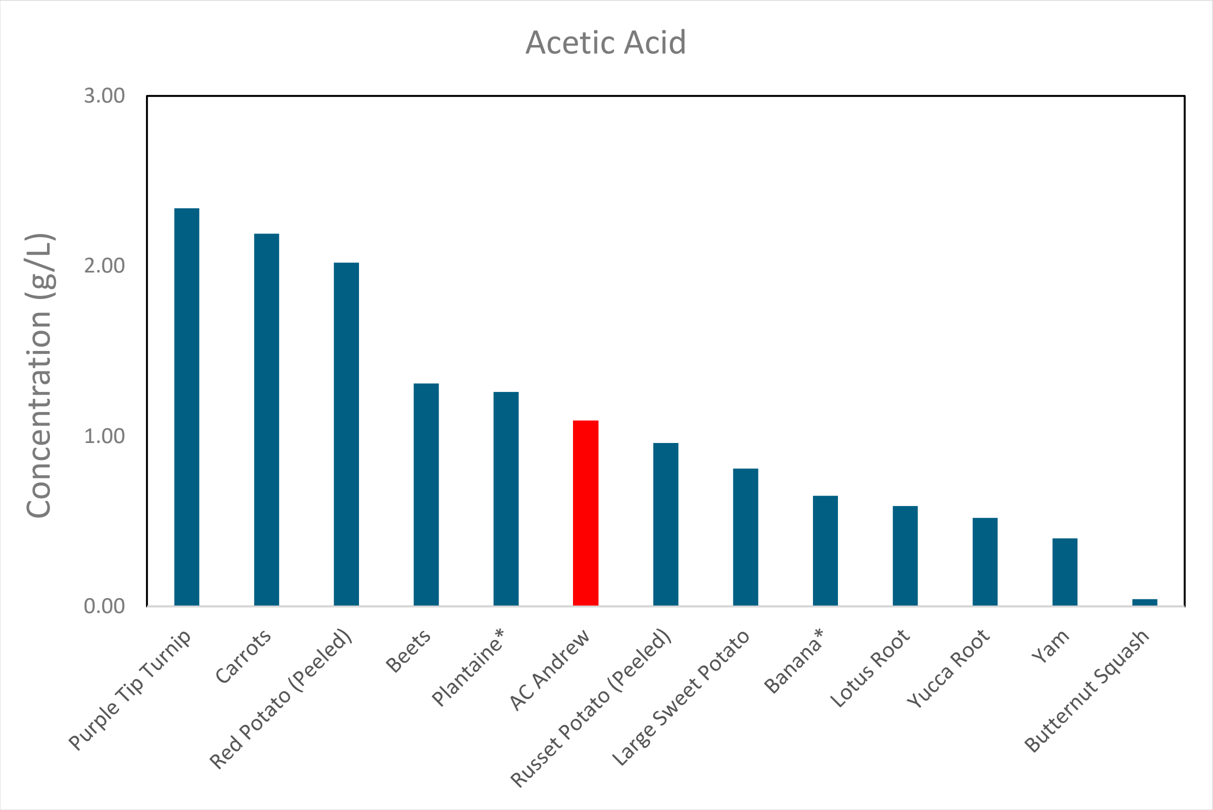

Figure S1. Average acetic acid concentrations, for each feedstock, after 72 h fermentation. Wheat is highlighted in red and other substrates as labelled in blue for comparative purposes.

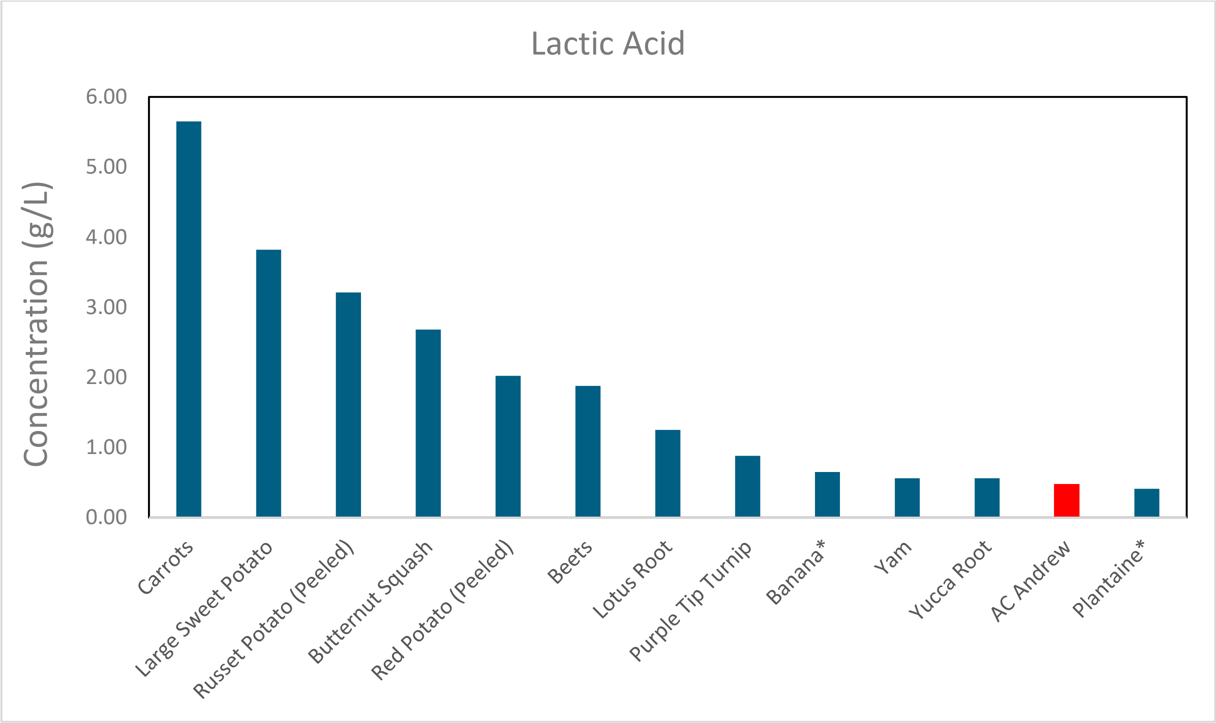

Figure S2. Average lactic acid concentrations, for each feedstock, after 72 h fermentation. Wheat is highlighted in red and other substrates as labelled in blue for comparative purposes.

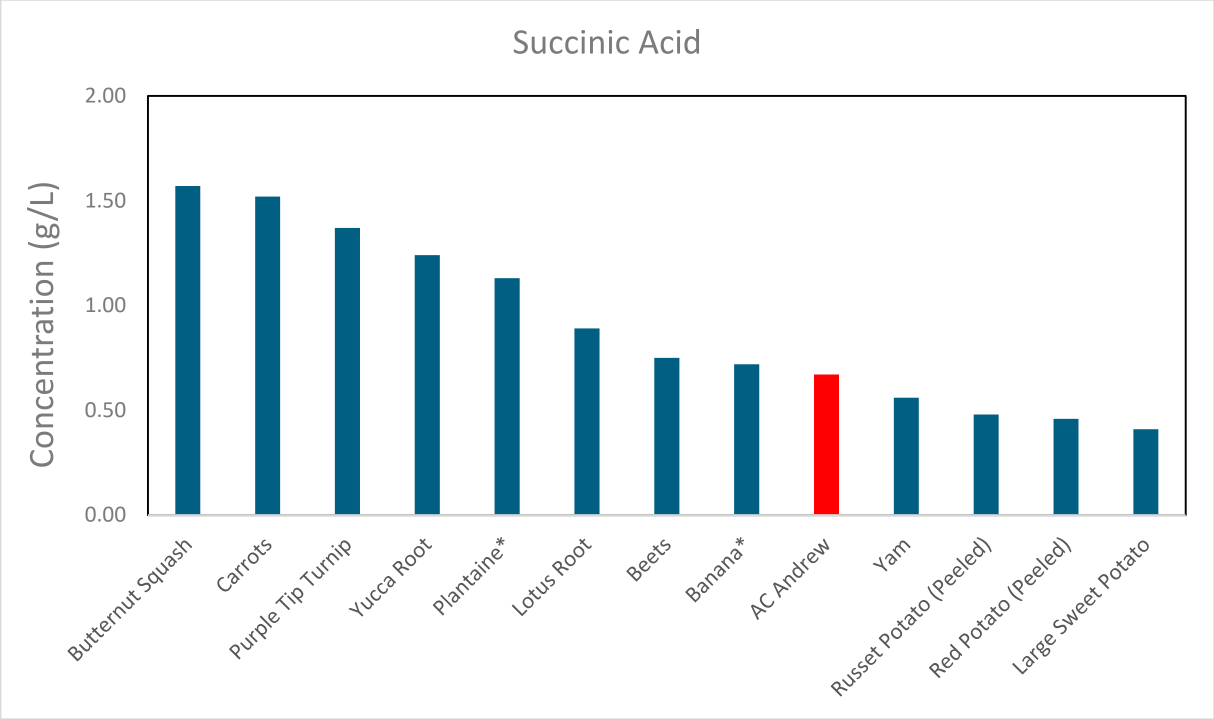

Figure S3. Average succinic acid concentrations, for each feedstock, after 72 h fermentation.

Wheat is highlighted in red and other substrates as labelled in blue for comparative purposes.

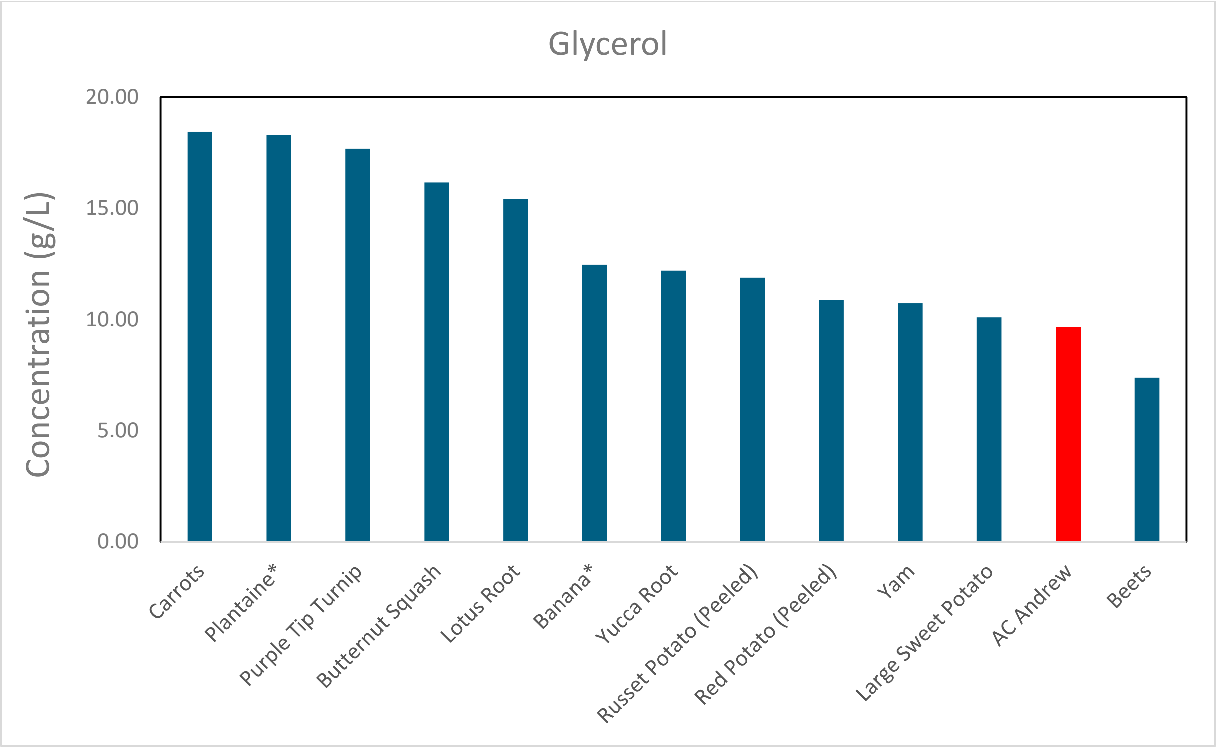

Figure S4. Average glycerol concentrations, for each feedstock, after 72 h fermentation. Wheat is highlighted in red and other substrates as labelled in blue for comparative purposes.
